# Factores asociados a la percepción de discriminación: edadismo y calidad de vida de la población geriátrica desde un enfoque bioético

**DOI:** 10.31053/1853.0605.v80.n3.38107

**Published:** 2023-09-29

**Authors:** Germán Brito Sosa, Ana María Iraizoz Barrios, Viviana García Mir, Jovanny Angelina Santos Luna, Gisela de los Ángeles León García, Raquel Magali Simbaña Jaramillo

**Affiliations:** 1 Universidad Técnica de Machala

**Keywords:** anciano, discriminación social, bioética, calidad de vida, ageísmo, aged, social discrimination, bioethics, quality of life, ageism, idoso, discriminação social, bioética, qualidade de vida, ageismo

## Abstract

**Introducción:**

En la vejez se presentan un conjunto de condiciones que afectan directamente la calidad de vida del adulto mayor, ocasionando marginación y discriminación.

**Objetivo:**

Determinar los factores biopsicosociales que inciden en la percepción de los adultos mayores de discriminación y calidad de vida en la provincia El Oro, Ecuador.

**Métodos:**

Estudio observacional, descriptivo, cualitativo-fenomenológico, de corte transversal, en adultos mayores de 65 años, entre septiembre del 2019 y noviembre del 2020. La muestra fue 399 adultos mayores. Se utilizó el cuestionario: "Valoración biopsicosocial del adulto mayor desde un enfoque bioético". Se midieron las variables: discriminación y autopercepción de calidad de vida. Se utilizó el análisis de correspondencia múltiple, para examinar la asociación entre discriminación, calidad de vida, y las variables en estudio.

**Resultados:**

De los adultos mayores encuestados, 246 (61,7% - IC 95% 0,58-0,66) consideraron que existe discriminación, predominando el edadismo. La variable más relacionada a la percepción de discriminación fue el trato en la atención sanitaria, y el entorno familiar. La calidad de vida en un porcentaje significativo fue insatisfactoria debido a: su entorno familiar, la poca integración social e insatisfacción con su salud.

**Conclusiones:**

Los principales factores biopsicosociales que los adultos mayores relacionan a su percepción de discriminación y edadismo fueron el trato en el entorno familiar y en los servicios de salud, incumpliéndose con los principios de la bioética. Los factores que influyen negativamente en la calidad de vida de los adultos mayores están relacionados con la familia, la integración social y el estado de salud.

## Abstract (en)

**Introduction:**

There are a set of conditions that directly affect the quality of life of older adults, causing marginalization and discrimination.

**Objective:**

Determining the biopsychosocial factors that affect the perception of discrimination and quality of life of older adults in the El Oro province, Ecuador.

**Methods:**

Observational, descriptive, qualitative-phenomenological, cross-sectional, cross-sectional study in older adults aged 65 years, between September 2019 and November 2020. The sample consisted of 399 older adults. The questionnaire "Biopsychosocial assessment of older adults from a bioethical approach" was used. The following variables were measured: discrimination and self-perception of quality of life. Multiple correspondence analyses were used to examine the association between discrimination, quality of life and the variables under study.

**Results:**

Of the older adults surveyed, 246 (61.7% - 95% CI 0.58-0.66) considered that discrimination exists, with ageism predominating. The variable most related to the perception of discrimination was the treatment in health care, and the family environment. The quality of life in a significant percentage was unsatisfactory due to: their family environment, poor social integration and dissatisfaction with their health.

**Conclusions:**

The main biopsychosocial factors that older adults relate to their perception of discrimination and ageism were treatment in the family environment and in health services, failing to comply with the principles of bioethics. The factors that negatively influence the quality of life of the elderly are related to the family, social integration and health status.

## Resumo

**Introdução:**

Na velhice existe um conjunto de condições que afetam diretamente a qualidade de vida do idoso, causando marginalização e discriminação.

**Objetivo:**

Determinar os fatores biopsicossociais que afetam a percepção dos idosos sobre discriminação e qualidade de vida na província de El Oro, Equador.

**Métodos:**

Estudo observacional, descritivo, qualitativo-fenomenológico, transversal em adultos com mais de 65 anos, entre setembro de 2019 e novembro de 2020. A amostra foi de 399 idosos. Utilizou-se o questionário: "Avaliação biopsicossocial do idoso sob o enfoque bioético". Foram mensuradas as variáveis: discriminação e autopercepção da qualidade de vida. A análise de correspondência múltipla foi utilizada para examinar a associação entre discriminação, qualidade de vida e as variáveis em estudo.

**Resultados:**

Dos idosos pesquisados, 246 (61,7% - IC 95% 0,58-0,66) consideram que existe discriminação, predominando o preconceito de idade. A variável mais relacionada à percepção de discriminação foi o tratamento na área da saúde e o ambiente familiar. A qualidade de vida em percentual significativo foi insatisfatória devido a: ambiente familiar, pouca integração social e insatisfação com a saúde.

**Conclusões:**

Os principais fatores biopsicossociais que os idosos relacionam com sua percepção de discriminação e preconceito de idade foram o tratamento no ambiente familiar e nos serviços de saúde, descumprindo os princípios da bioética. Os fatores que influenciam negativamente a qualidade de vida dos idosos estão relacionados com a família, a integração social e o estado de saúde.

**CONCEPTOS CLAVE**

Qué se sabe sobre el tema: Edadismo o ageismo es un término que hace referencia a los estereotipos, prejuicios, y discriminación hacia las personas por su edad. En los últimos años se ha incrementado la discriminación contra los adultos mayores.

Qué aporta este trabajo: El presente estudio mostró que los adultos mayores de la provincia perciben discriminación, principalmente edadismo, en el trato en la atención de salud y en el entorno familiar, y manifiestan insatisfacción con su calidad de vida.

**Divulgación**

En los últimos años y particularmente en la situación de pandemia, se ha evidenciado un aumento de la discriminación a las personas de la tercera edad, quienes son maltratados, rechazados o excluidos por diferentes sectores de la sociedad, vulnerando sus derechos. Esta situación, más las desventajas propias del envejecimiento donde el desgaste físico y mental afectan las relaciones sociales, perturban el bienestar y la esperanza de vida de los ancianos. En el presente trabajo nos propusimos determinar aquellos factores biopsicosociales que pudieran afectar la calidad de vida de los adultos mayores al hacerlos sentir discriminados, ya sea por la edad u otra causa. Para ello se estudió una muestra de la población mayor de 65 años de la provincia El Oro, Ecuador, encontrándose que efectivamente apreciaban discriminación, fundamentalmente por la edad, en la atención de salud y el entorno familiar específicamente, lo que afecta su salud y bienestar.

## Introducción

En la vejez se presentan un conjunto de condiciones sociales, económicas, familiares, y culturales interrelacionadas, que afectan directamente la calidad de vida (CV) del adulto mayor (AM), ocasionando la marginación y discriminación de este grupo etario ^1^ .

La Organización Mundial de la Salud (OMS) define la calidad de vida como "la percepción que tiene un individuo de su posición en la vida, en el contexto de la cultura y los sistemas de valores en los que vive y en relación con sus metas, expectativas, estándares e inquietudes" ^2^ . En los AM la CV depende de la salud biológica, y del buen funcionamiento mental, social, cultural, y espiritual ^3^ .

Ante el aumento de la esperanza de vida a nivel mundial, muchos países han realizado modificaciones en los programas sociales y de salud, encaminados a mejorar la CV ^4^ . Los pronósticos actuales indican que los AM de 60 años se incrementarán de 900 millones en el 2015, a 1400 millones para el 2030, y a 2100 millones para el año 2050, por lo que se torna necesario e imprescindible encaminar las políticas sociales y de salud a un envejecimiento activo, funcional y saludable, que permita a este grupo etario una vida digna ^5 6^ .

Con el surgimiento de la ética médica, aparece una disciplina en la medicina que vincula la atención sanitaria con la ética, ciencia filosófica que estudia la moral ^7 8^ . Con el surgimiento de la bioética, las ciencias de la salud se vinculan con otras disciplinas favoreciendo su desarrollo, con mayor posibilidad de soluciones.

En el año 2020 ante el edadismo (estereotipos, prejuicios, y discriminación por razones de edad) evidenciado durante la pandemia por el COVID-19 en la atención de salud a los AM, la OMS lanzó una iniciativa cuyo objetivo es mejorar la percepción de la comunidad acerca del envejecimiento, y combatir la discriminación etaria. Esta iniciativa forma parte integral de la Década de Envejecimiento Saludable de las Naciones Unidas (2021-2030) ^9 10^ .

En la provincia El Oro, Ecuador, no se ha indagado acerca de si existen factores que pudieran hacer experimentar a los AM edadismo u otros tipos de discriminación. Por tanto, en el presente estudio nos hemos propuesto determinar los factores biopsicosociales que inciden en la percepción de los AM de discriminación, y afectan su CV.

## Metodología

### Diseño de investigación y contexto

Se realizó un estudio descriptivo, observacional y de corte transversal en AM de la provincia de El Oro, Ecuador, entre septiembre del 2019 y noviembre del 2020. La provincia se encuentra situada al sur del país, es fronteriza con Perú.

### Participantes

En el último censo poblacional la cantidad de AM de la provincia se estimó para el año 2020, en 53 852 habitantes ^11^ .

Fueron incluidos en la investigación AM de 65 años o más, que vivían en la provincia, y aceptaron participar. Fueron excluidos los AM que presentaban estados avanzados de demencia senil, incapacidad visual severa o de otra índole, que impedían realizar adecuadamente la encuesta, así como aquellos que se negaron a firmar el consentimiento informado.

Se calculó el tamaño de la muestra en base a la fórmula de poblaciones finitas con un 5% de error muestral y 95% de nivel de confianza, quedando constituida por 399 AM. El muestreo fue probabilístico, aleatorio simple, sin reemplazo.

Estudiantes de la carrera de Enfermería de la Universidad Técnica de Machala (UTMACH) previamente capacitados en la aplicación del cuestionario por profesores del grupo de investigación "Adulto mayor y bioética", recopilaron la información al realizar las entrevistas a los AM en sus casas, de forma individual, cuidando la privacidad para facilitar las respuestas sin restricciones.

### Instrumento y variables

El instrumento de la investigación fue el cuestionario: "Valoración biopsicosocial del AM desde un enfoque bioético", encuesta validada por un comité de expertos integrado por psicólogos y geriatras en Cuba en el año 2012. Este comité revisó la claridad, coherencia, relevancia y suficiencia del instrumento, realizando una prueba piloto que permitió corregirla, antes de ser aplicada a la población cubana ^12^ . En Ecuador se realizó una prueba piloto en el Hogar de Ancianos Esteban Quirola de la ciudad de Machala, para corregir cualquier dificultad idiomática, y ajustarla a las características regionales.

Este cuestionario consta de 20 ítems, a los que se adicionaron dos preguntas relacionadas con: la satisfacción con el estado de salud, y autopercepción de calidad de vida (preguntas 21 y 22). De la pregunta 1-4 se recoge información referentes al estado de salud del encuestado (enfermedades que padece, hábitos nocivos, estilos de vida saludables o no, ingestión de medicamentos), de la 5-8 para valorar la asistencia médica (necesidad de asistencia médica, frecuencia, motivo), las preguntas de la 9 - 13 y la pregunta 19, se utilizaron para determinar cómo se sienten tratados en su medio familiar (trato y apoyo familiar, sentirse útil, privacidad, autoridad, y capacidad de atenderse a sí mismo o autocuidado). La 14 y la 15 para identificar sus principales preocupaciones y detectar si existe discriminación hacia el adulto mayor respectivamente, y de la 16 a la 20, excluyendo la 19, para realizar un análisis de su integración a la sociedad (participación en círculos o club sociales, recluido en hogar de anciano, soledad, autopercepción de integración social) ^12^ .

En la evaluación de la fiabilidad de la consistencia interna de los ítems se obtuvieron valores adecuados de coeficientes: alfa de Cronbach de 0,739 (superior a 0,70) y omega de McDonald de 0,804. Para facilitar el procesamiento de los datos de los análisis multivariados, se reformularon las opciones de respuestas, siendo la mayoría dicotomizadas. Por tanto, se evaluó nuevamente la consistencia interna obteniendo un coeficiente Kuder-Richardson (KR20 -Horst) de 0,84 ^13^ .

Las variables sociodemográficas consideradas en este estudio fueron: la edad, género, estado civil y estado laboral. La edad se agrupó, obteniéndose rangos de 65 - 68, 69 - 76 y 77 - 98 años. Los adultos mayores casados y convivientes fueron incluidos en la categoría "Con Pareja" y los solteros, separados y viudos, en la categoría "Sin Pareja" (variable pareja sentimental). Del mismo modo, se dicotomizaron las variables capacidad laboral (activo, trabajando) y enfermedad al momento de la recogida de datos, en sí o no. Las variables principales de este estudio fueron la percepción de discriminación (sí o no), y calidad de vida (satisfactoria o insatisfactoria).

### Análisis estadístico

Se utilizó el paquete estadístico SPSS, versión 25 para Windows. Se empleó estadística descriptiva univariada, bivariada, y multivariada. De las variables cualitativas se obtuvieron frecuencias absolutas y porcentajes, y en las cuantitativas se usaron medidas de tendencia central y dispersión, incluyendo los intervalos de confianza (IC). La relación de variables se evaluó con la prueba estadística Chi-Cuadrado, como paso previo para realizar el análisis de correspondencia múltiple (ACM). Las posiciones relativas de los puntos en la representación geométrica de los resultados en el ACM, permitieron establecer un criterio global de los niveles de similitud o asociación entre las variables y las categorías. Los valores de p menores a 0.05 se consideraron significativos.

### Aspectos éticos

La investigación realizada fue aprobada por el Comité de Ética de la Universidad Técnica de Machala. Los adultos mayores encuestados firmaron el consentimiento informado, donde se explicaba el objetivo e importancia del presente estudio. Se garantizó el anonimato y la confidencialidad de los datos.

## Resultados

El estudio realizado quedó constituido por 399 AM: 229 mujeres (57,4%, IC 0,52-0,62), y 170 hombres (42,6%). El rango de edades estaba entre 65 y 98 años con una media de 73,7 ± 7,4 años (mediana: 73). Realizaban poca actividad física 138 AM (34,6% IC 0,32-0,36). Vivían con sus parejas 185 de los AM (46,4%, IC 0,41-0,51), y 109 eran viudos (27,3%). La prevalencia de enfermedades se constató en 305 AM (76,4%, IC 0,72-0,81), de las cuales 181 eran mujeres (45,3%, IC 95% diferencia -0,14 - -0,09). La hipertensión arterial y en general las enfermedades cardiovasculares fueron las enfermedades de mayor prevalencia, seguidas por las osteomusculares y las endocrinas. Eran pluripatológicos 182 AM (45,6%, IC 0,41-0,50).

Un total de 249 AM (62,4%, IC 0,61-0,70) refirieron que acudían para su asistencia médica a instituciones públicas (subcentros y hospitales), y 150 AM (37,6%) a instituciones privadas. El 59,7% de los AM necesitaban asistencia médica con una frecuencia menor a 3 meses, el 26,1% antes de los 6 meses y el 14,2% anual.

A continuación se muestra la percepción del AM de discriminación, donde se evidencia que el 61,7% se sienten discriminados, constituyendo el edadismo la causa más frecuente (Figura 1).

**Figura N°1.**
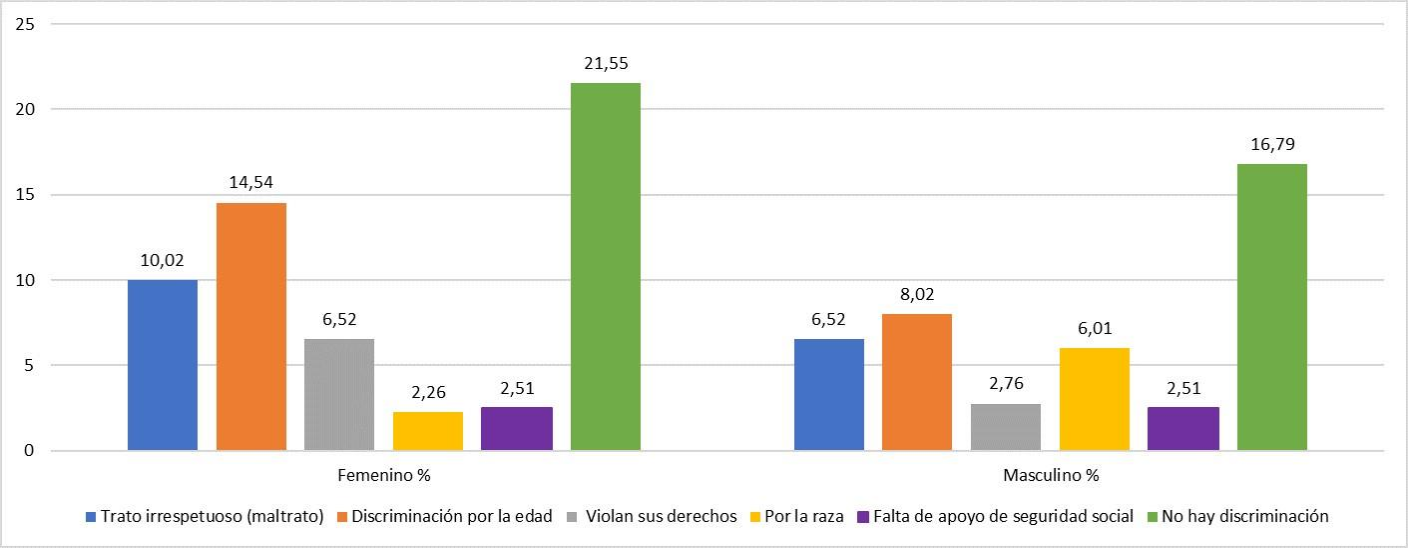
Principales razones aportadas por los encuestados acerca de la percepción de discriminación, según el género.

Mediante la prueba chi cuadrado se determinó que no existía asociación entre la discriminación y las variables: género, pareja sentimental, capacidad laboral, y enfermedad, obteniendo valores de significación p >0.05.

De los 159 (39,8 %) AM que reconocen haber tenido un trato inapropiado en la atención sanitaria, el 93,7% consideran que existe discriminación con el AM por las siguientes causas: la atención del personal sanitario es deficiente y con poca empatía, en ocasiones algunos médicos y enfermeras han tenido un trato deplorable, no les dan prioridad al AM, no son tomados en cuenta por la edad, no son tomados en cuenta por la gravedad, no disponen de seguro social, entre otras (Tabla 1).

**Tabla N°1:** Relación entre las principales variables en estudio y la percepción de discriminación

Variables	Percepción de discriminación						p
	Si		No		Total		
	246 (61,7%)		153 (38,3%)		399 (100%)		
	n	%	n	%	n	%	
Trato en la atención de salud							
Bien	97	24,3	143	35,8	240	60,2	<0.001
Regular	84	21,1	5	1,3	89	22,3	
Mal	65	16,3	5	1,3	70	17,5	
Trato familia	
Satisfactorio	172	43,1	150	37,6	322	80,7	<0.001
Insatisfactorio	74	18,6	3	0,7	77	19,3	
Apoyo familiar							
Si	175	43,9	146	36,6	321	80,5	<0.001
No	71	17,8	7	1,7	78	19,5	
Se siente útil en su medio familiar							
Si	182	45,6	149	37,4	331	82,9	<0.001
No	64	16,1	4	1	68	17,1	
Capacidad de atenderse a sí mismo							
Si	191	49,9	122	30,5	313	78,4	0.621
No	55	13,8	31	7,8	86	21,6	
Integración social							
Si	220	55,1	137	34,3	357	89,4	0.557
No	26	6,5	16	4	42	10,5	
Respetan su privacidad							
Si	213	53,3	144	36,1	357	89,47	0.017
No	33	8,3	9	2,2	42	10,5	
Pérdida de autoridad							
Nunca	145	36,3	73	18,3	218	54,6	0.059
En ocasiones	66	16,5	47	11,8	113	28,3	
Frecuentemente	35	8,8	33	8,3	68	17,1	

Los AM que no se sienten bien tratados por sus familiares, consideran que existe discriminación porque la familia: no los ayudan, no los apoyan, no los visitan, no los toman en cuenta, no se preocupan por su salud, por razones económicas, problemas personales, por ser mayores, y estar enfermos.

De los 78 AM (IC 95% 0,16-0,24) que consideraron no haber tenido el apoyo necesario de sus familiares, el 91% (IC 95% diferencia -0.31- -0.18) se sintieron discriminados porque: no los visitan, no se sienten que son tomados en cuenta, no los ayudan, ni apoyan económica ni emocionalmente, no se preocupan por su salud, y los abandonan.

Un porciento de los AM no se sienten útil en su medio familiar (n=68, 17 %, IC 0,14-0,21) porque: no son tomados en cuenta ni los incluyen en las actividades, no los visitan, son una carga para la familia por las limitaciones propias de la edad, no se pueden cuidar solos, ni pueden ayudar con la economía.

Los motivos por los cuales los AM consideran que no son capaces de atenderse a sí mismo se deben a: las enfermedades y limitaciones propias de la edad, no poder deambular, disminución de la agudeza visual, dificultad para alimentarse, pérdida de la memoria, disminución de la fuerza, entre otras. Los AM que refirieron no se consideraban capaces de mantenerse integrado a la sociedad fueron debido a: problemas de salud, edad avanzada, y discriminación.

Al analizar las variables demográficas con la CV se encontró asociación entre la CV y: el género (p=0.017), edad agrupada en intervalos de 68, ≤ 69-76 y ≥ 77 (p<0.001), y pareja sentimental (p<0.001); pero no con el hecho de padecer alguna enfermedad (p=0.079).

Al relacionar la percepción de la CV del AM con el resto de las variables, se encontró que todas muestran una relación significativa (p < 0.001) utilizando la prueba estadística chi cuadrado, menos el trato en la atención de salud (Tabla 2).

**Tabla N°2:** Relación entre las principales variables en estudio y la percepción de calidad de vida

Variables	Percepción de calidad de vida						p
	Satisfactoria 281 (70,4%)		Insatisfactoria 118 (29,6%)		Total		
					399 (100%)		
	n	%	n	%	n	%	
Trato en la atención sanitaria							
Bien	170	42,6	70	15,5	240	60,1	0.739
Regular	60	15,03	29	7,26	89	22,3	
Mal	51	12,8	19	4,8	70	17,6	
Trato familia							
Satisfactorio	263	65,9	59	14,8	322	80,7	<0.001
Insatisfactorio	18	4,5	59	14,8	77	19,3	
Apoyo familiar							
Si	263	65,9	58	14,5	321	80,4	<0.001
No	18	4,5	60	15,1	78	19,6	
Sentirse útil en su medio familiar							
Si	263	65,9	68	17	331	82,96	<0.001
No	18	4,5	50	12,5	68	17	
Capacidad de atenderse a sí mismo							
Si	234	58,6	79	19,8	313	78,4	<0.001
No	47	11,8	39	9,8	86	21,6	
Integración social							
Si	267	66,9	90	22,6	357	89,5	<0.001
No	14	3,5	28	7	42	10,5	
Respetan su privacidad							
Si	264	66,2	93	23,3	357	89,5	<0.001
No	17	4,2	25	6,3	42	10,5	
Pérdida autoridad							
Nunca	182	45,6	36	9	218	54,6	<0.001
En ocasiones	62	15,5	51	12,8	113	28,3	
Frecuentemente	37	9,3	31	7.8	68	17,1	
Satisfacción con el estado de salud							
No	56	14	103	25,8	159	39,8	<0.001
En ocasiones	133	33,3	10	2,5	143	35,8	
Si	92	23,1	5	1,2	97	24,3	

En el ACM de la variable discriminación (Figura 2) se observa que, si bien existe relación entre las variables en estudio, la variable más relacionada a la percepción de discriminación de los AM fue el trato en la atención sanitaria. El α_1_Cronbach del análisis fue 0,895 y α_2_ Cronbach 0,768 con una media de 0,848 aportando más la dimensión 1 a los resultados.

**Figura N° 2.**
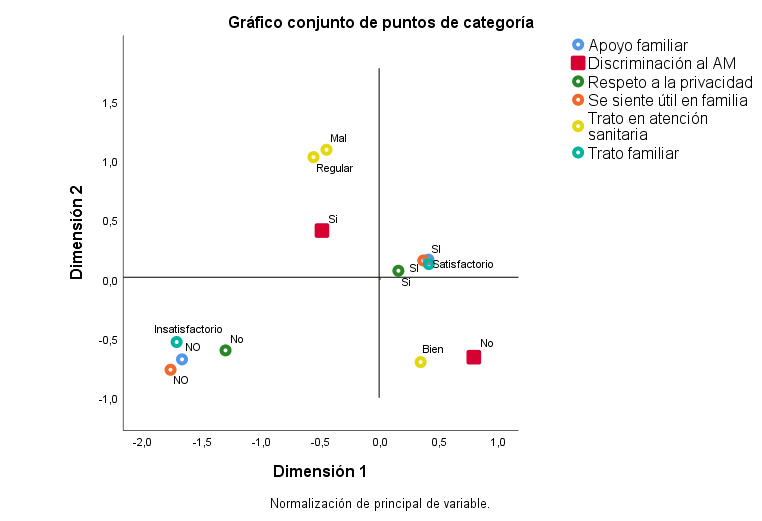
Representaciones gráficas del análisis de correspondencia donde se relacionan las variables significativas del estudio con la discriminación.

En el ACM de la variable CV (Figura 3) se observa la relación estrecha de la CV satisfactoria con la mayoría de variables en estudio. Se advierte una influencia significativa de las variables positivas relacionada con la familia (trato familiar, apoyo familiar, privacidad, la autoridad, el ser útil en el medio familiar), la integración social, el presentar pareja sentimental, el poder realizar su autocuidado, más que la variable relacionada con la presencia o no de enfermedades. Las edades más jóvenes entre los AM también reflejaron una mejor CV. El α_1_ Cronbach del análisis fue 0,908 y α_2_ Cronbach 0,775 con una media de 0,865 aportando más también la dimensión 1 a los resultados.

**Figura N° 3.**
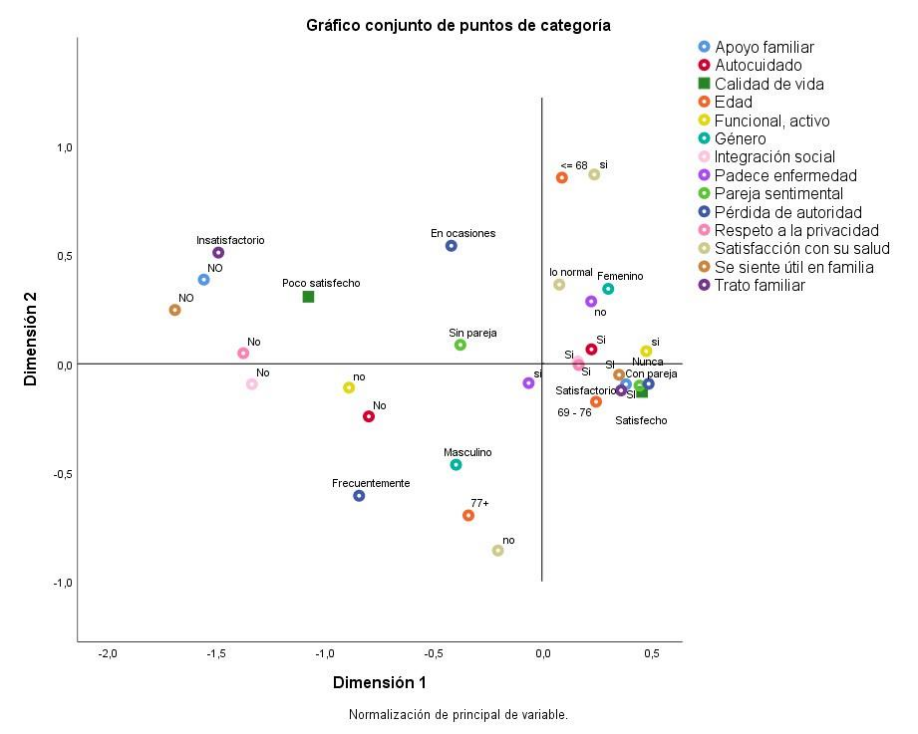
Representaciones gráficas del análisis de correspondencia donde se relacionan las variables significativas del estudio con la calidad de vida.

## Discusión

En el presente estudio nos propusimos determinar los factores biopsicosociales que inciden en la percepción de los AM de discriminación y CV en la provincia El Oro. Se pudo constatar la percepción de discriminación en una proporción representativa de los AM, siendo el motivo más frecuente el edadismo, y relacionada con el trato en la atención de salud, y por parte de sus familiares. En relación a la CV de los AM los factores biopsicosociales asociados fueron contar con una pareja sentimental, tener capacidad laboral, el trato familiar adecuado, y la satisfacción con su estado de salud.

Algunos estudios han demostrado la existencia de discriminación por edad ^6 15-19^ . Jackson et al. ^17^ en su estudio con 7731 encuestados encontró edadismo en el 25,1%, prevaleciendo frente a otros tipos discriminación, resultados que coinciden con nuestro estudio al percibir edadismo en el 22,6%. Browning et al. ^19^ confirmaron la asociación negativa entre las percepciones de la discriminación por edad, los niveles de salud mental, y la satisfacción con la vida.

La discriminación por motivos de edad actualmente, es una forma más generalizada de discriminación que el sexismo o el racismo. Al sembrar en el AM los estereotipos del aislamiento social, el deterioro físico y cognitivo, la falta de actividad física, y la idea de que son una carga económica, puede convertirse en realidad ^6 20-22^ .

La mayoría de los AM que tuvieron un trato sanitario inadecuado consideran existe discriminación. Para revertir esta situación es importante abogar por que el sistema de salud pase a ser un elemento central en la protección social y reducción de las inequidades, contribuyendo en gran medida a la cohesión, la justicia y el bienestar social. Es aconsejable, además, educar y formar a los profesionales de la salud en el conocimiento y aplicación de los principios bioéticos, para que puedan ofrecer una atención humanizada ^3 16 23 24^ .

Las variables estudiadas relacionadas con el entorno familiar han influido en la percepción de discriminación y la CV del AM. En más del 80% de la población encuestada, el entorno familiar ha constituido un gran apoyo, pero en un porciento con significación p<0.001, el AM ha sentido discriminación en su seno familiar, influyendo negativamente en su CV. En diversos estudios se ha demostrado la existencia de discriminación del AM por sus familiares, fundamentalmente por negligencia, edadismo, violencia psicológica, abuso económico, entre otros maltratos ^25^ . La familia debe apoyar y respetar las necesidades, preferencias, los valores, y la autonomía del AM para contribuir con un envejecimiento exitoso ^26^ .

La percepción de integración social fue favorable en más del 80% de los AM, sin embargo, en otros estudios se ha evidenciado lo contrario, encontrando discriminación social del AM ^15^ .

La autopercepción de CV en los AM estudiados fue satisfactoria en más del 70%, aunque en otros estudios ^27^ ha sido deficiente en más de 45%, afectando en mayor proporción: a las mujeres, personas con menor escolaridad, y a medida que se incrementa la edad. Aproximadamente el 60% de los AM consideran tener satisfacción con su estado de salud, porciento similar al encontrado por Flores et al. ^27^ de 63,7%, aunque, menor que en el estudio realizado por Benavente et al. ^6^ , donde la percepción óptima de salud fue mayor al 80%. Los resultados suelen ser diversos en los diferentes estudios realizados, aunque la percepción de la CV del AM estará influenciada por al apoyo familiar, la integración social y con la satisfacción de su estado de salud, variables que son predictivas de la CV. También influye la habilidad de adaptación del AM en el aspecto social, físico y emocional ^28^ .

Los AM tienen el mismo derecho que todos a tener un trato sin diferencias, ni discriminación, como afirma el principio de la justicia ^3^ . Uno de los pilares básicos del envejecimiento es lograr la preservación de la autonomía tanto física como psicológica, y debe ser estimulado en el AM para promover la autodeterminación, y la dignidad que da responsabilidad y sentido a la dimensión ética de la salud. Hernán L. Fuenzalida-Puelma considera que la bioética es una herramienta útil para mejorar la CV, e incrementar la capacidad del desarrollo individual y comunitario. Por tener la bioética una relación directa con los derechos humanos, defendidos con carácter universal ^29^ , y constituir un recurso para la formación de políticas públicas con impacto en el mundo social ^30^ , sería de gran utilidad: promover programas comunitarios dirigidos a integrar a los AM y mejorar la imagen que la persona adulta mayor tiene de sí misma, la familia y la sociedad; promover estilos de vida, hábitos y costumbres que favorezcan la salud; desarrollar nuevos procesos de investigación relacionados con la Geriatría y Gerontología en coordinación interinstitucional e intersectorial ^7^ .

En este estudio se identificaron limitaciones propias de la investigación cualitativa, como el tamaño de la muestra y el muestreo probabilístico aleatorio simple, sin establecer diferencias entre sectores de la población, o hacer estratificación. Adicionalmente se trata de un estudio transversal donde se utilizó un cuestionario de autoinforme, por lo que recomendamos realizar estudios longitudinales y utilizar métodos tanto cuantitativos como cualitativos. Algunos resultados además pueden estar influenciados, por ejemplos que no se haya encontrado una relación significativa de la calidad de vida con la presencia o no de enfermedades puede estar influenciado por el hecho de que no se clasificó en base a la severidad o tipo de enfermedades que padece. No obstante, constituye un primer acercamiento a una realidad internacional en esta región, y la identificación de que existe necesidad social de crear estrategias e intervenciones factibles para reducir la discriminación por edad.

En conclusión, los principales factores biopsicosociales que los adultos mayores relacionaron a su percepción de discriminación y edadismo fueron el trato en el entorno familiar y en los servicios de salud, incumpliéndose con los principios de la bioética. Los factores que influyen negativamente en la calidad de vida de los adultos mayores están relacionados con la familia, la integración social y el estado de salud. Con estos resultados consideramos que el factor que más está afectando el bienestar de los adultos mayores es el social, lo cual debería seguir estudiándose para mejorar las políticas sociales y de salud. Se recomienda enfáticamente realizar investigaciones que realicen intervenciones educativas efectivas para reducir la discriminación por edad.
